# Disparities in dialysis allocation: An audit from the new South Africa

**DOI:** 10.1371/journal.pone.0176041

**Published:** 2017-04-18

**Authors:** Kajiru G. Kilonzo, Erika S. W. Jones, Ikechi G. Okpechi, Nicola Wearne, Zunaid Barday, Charles R. Swanepoel, Karen Yeates, Brian L. Rayner

**Affiliations:** 1 Division of Nephrology and Hypertension, Department of Medicine, Faculty of Health Sciences, University of Cape Town, Cape Town, Western Cape, South Africa; 2 Internal Medicine Department, Kilimanjaro Christian Medical University College, Kilimanjaro, Moshi, Tanzania; 3 Department of Nephrology, Queen’s University, Kingston, Ontario, Canada; Postgraduate Medical Institute, INDIA

## Abstract

End Stage Kidney Disease (ESKD) is a public health problem with an enormous economic burden. In resource limited settings management of ESKD is often rationed. Racial and socio-economic inequalities in selecting candidates have been previously documented in South Africa. New guidelines for dialysis developed in the Western Cape have focused on prioritizing treatment. With this in mind we aimed at exploring whether the new guidelines would improve inequalities previously documented. A retrospective study of patients presented to the selection committee was conducted at Groote Schuur Hospital. A total of 564 ESKD patients presented between 1 January 2008 and 31 December 2012 were assessed. Half of the patients came from low socioeconomic areas, and presentation was late with either overt uremia (n = 181, 44·4%) or fluid overload (n = 179, 43·9%). More than half (53·9%) of the patients were not selected for the program. Predictors of non-acceptance onto the program included age above 50 years (OR 0·3, p = 0·001), unemployment (OR 0·3, p<0·001), substance abuse (OR 0·2, p<0·001), diabetes (OR 0·4, p = 0·016) and a poor psychosocial assessment (OR 0·13, p<0·001). Race, gender and marital status were not predictors. The use of new guidelines has not led to an increase in inequalities. In view of the advanced nature of presentation greater efforts need to be made to prevent early kidney disease, to allocate more resources to renal replacement therapy in view of the loss of young and potentially productive life.

## Introduction

Chronic kidney disease (CKD) is a global public health problem. Globally and in developing countries, the burden of CKD is predicted to worsen.[1] The major risk factors in Sub Saharan Africa (SSA) include hypertension, diabetes, HIV and glomerulonephritis. [2–4] Prevention of CKD remains inadequately addressed due to resource limitation.[5,6] Treatment of end stage kidney disease (ESKD) in SSA is severely limited or non-existent.[2,5,7] For patients with ESKD, dialysis and transplantation is a life sustaining option. Dialysis programs for indigent patients are by necessity forced to ration dialysis often favoring the most suitable candidates while denying life prolonging therapies to others.

South Africa represents a sustainable model of dialysis rationing. The need for rationing is evident. South Africa has 2·1 nephrologists per million population as compared with 16 nephrologists per million population in the United States.[8] Furthermore, it is estimated that about 600 to 2000 indigent patients are left untreated annually in the Western Cape.[9] In the context of resource constraints, the National department of Health in 1997 introduced dialysis and transplantation guidelines which emphasized equitable access to treatment.[10] Discussing guidelines based on equity was justified given the data available at that time. In the year 1994 the overall treatment acceptance rate in South Africa was 17 patients per million population per year (PPM/year). Blacks (8·4 PPM/year) were under represented compared to mixed ancestry (32 PPM/year), White (41 PPM/year), or Asians (97 PPM/year).[11] The dangers of rationing dialysis were further demonstrated in one Western Cape hospital whereby patients likely to be selected were young and white.[12] Newer guidelines were published in 2010 by the Department of Health, Western Cape that prioritized selection on the basis of suitability for transplantation. This work explores the outcome of rationing dialysis in a public hospital in the Western Cape to determine if inequities in the selection process were still present.

## Materials and methods

This was a retrospective analytic study in which characteristics and allocation outcomes of patients assessed by the renal replacement therapy (RRT) committee at Groote Schuur. Groote Schuur is a state owned and tertiary hospital serving the city of Cape Town. In 2011, the population in the city of Cape Town was 3·7 million[13] with the following ethnic distribution: Black Africans 38·6%, Mixed 42·4%, Asians 1·4%, Whites 15·7% and others 1·9%. In the Western Cape prior to 1994 Blacks were more severely disadvantaged than Whites, Mixed Ancestry and Asian patients. Because of the few numbers of Whites in the audit, Whites, Mixed ancestry and Asians were group together as non-Blacks.

All new patients presented to the RRT Committee at the Groote Schuur Hospital from January 1^st^ 2008 to December 31^st^ 2012 were included in the audit.

The composition of a RRT committee included the referring doctor, social worker, at least 2 Nephrologists, nursing staff and the medical manager of the hospital. The Department has an allocation of 148 (plus 4 Hepatitis B positive patients) slots for treating ESKD patients. Assessments were limited to patients with CKD 5.

The assessment of eligibility for the renal replacement program was based on the information made available to the committee by the referring doctor and social worker. Poor psychosocial assessment was performed by the social worker based on a constellation of findings.

Patients were classified into three categories based on a renal assessment guidelines developed in 2010.[9]The following prioritization categories were used to judge acceptance onto the program. Category 1 patients were deemed most suitable for transplantation and would always be accommodated. Category 3 patients were those where transplantation was contra-indicated or associated with poorer outcomes, and these patients received optimal conservative care. Category 2 patients were patients with significant co-morbidities for example diabetes or HIV where transplantation could be undertaken but with higher costs and higher morbidity. These patient would be offered dialysis only if there were available slots. Before 2010, patients were accepted onto the renal replacement program based on their transplantability and no prioritization was made. For example HIV positive patients were generally excluded because transplantation was considered contra-indicated. This changed after the development of a HIV positive transplant programme in 2009. A comparison of the guidelines is presented in Table 1. To be able to compare the period before and after introduction of the new guidelines, patients were re-classified according to the new guidelines. This means that the same criteria to categorize patients were used in all periods.

**Table 1:** Comparison of Western Cape Guidelines (2010) with the Department of Health Guidelines (2009).

Aspect	Western Cape Guidelines[9], 2010	Department of Health Guidelines[15], before 2010
Principles	Patients must be suitable for transplantation	Transplantation a major criterion
	Guide on modality of chronic dialysis not stated.	Patient and family should be allowed to choose the modality of chronic dialysis
Selection criteria	Both inclusion and exclusion criteria used for selection	Exclusion rather than inclusion criteria applied for selection
Medical	Medical exclusion criteria include active malignancy and advanced irreversible progressive disease of vital organs	Medical exclusion criteria include active malignancy and advanced irreversible progressive disease of vital organs
	Diabetes will be considered below the age of 50years. Comorbid diseases may be considered.	Diabetes and acceptable comorbidity may be considered
	Hepatitis B e Antigen positivity to be excluded	Hepatitis B e Antigen Not specified
	Morbid obesity BMI>35 to be excluded	BMI limits not specified
	Age above 60 year are excluded	No age limit stated
	HIV +ve with undetectable viral load and CD4 count > 200 on 6 months of antiretrovirals	
Psychosocial	Mental illness with diminished functional capacity as shown by psychiatric and medical examination.	Mental illness with diminished functional capacity
	Habitual non adherence with any medical treatment	Habitual non-compliance with dialysis treatment and lifestyle modification.

The protocol had ethical approval from the Faculty of Health sciences, Human Research Ethics Committee of the University of Cape Town (HREC REF: 085/2013). Consent was not obtained in view of the retrospective design and the fact that investigators were part of the treating team.[14]

### Data collection and management

Patient’s characteristics and the outcome of the RRT committee were derived from the outcome letter, record of proceedings and psychosocial assessments. Additional socio-demographic information was obtained from Groote Schuur Hospital Clinicom database. Data from the newly compiled case record form was then entered into Statistical Package for Social Sciences (SPSS) v22·0·0·0.

### Data analysis and statistical considerations

Data cleaning was performed by two methods. Firstly, by manually comparing the data with the meeting proceedings books, the data were checked for accuracy. Secondly, each variable was re-checked for errors using descriptive statistics and scatterplots in SPSS.

The dataset was summarized into cross tables and bar charts to describe the study population in three separate time periods: 2008 to 2009 (when no prioritization guidelines were used), 2010 (when new guidelines were being introduced but some patients were still assessed using the old guidelines) and, 2011 to 2012 (when the new guidelines were used consistently).

Univariate analysis was performed to assess factors associated with acceptance onto the RRT program. Chi square and odd ratios were calculated. The factors which were found to be significant were then assessed using logistic regression (multivariate analysis). In both instances (univariate and multivariate analysis), a p-value of less than 0·05 was considered as significant.

## Results

Between January 1^st^, 2008 and December 31^st^, 2012 there were 564 new assessments of which 280 (49.6%) patients were rejected (category 3). he remainder included 212 (37.6%) patients who were placed on the waiting list (category 2) and 72 (12.8%) patients were accepted (category1). The ethnic distribution was Black 43.1% (n = 243), whites 5.14% (n = 29), Mixed 51.24 (n = 289) and Asian 0.53% (n = 3). The baseline characteristics of Blacks vs. Non-blacks is presented in Table 2.

**Table 2:** Baseline demographics of Blacks vs. Non-Blacks^+^ among new patients presented to the renal assessment committee in Groote Schuur from 2008–2012.

Variable (N**)	Race			Χ^2^	p-value*
	Blacks N (%)	Non-Blacks N (%)	All N (%)		
Male gender (564)	146(60·1)	172(53·6)	318(56·4)	2·38	0·12
Age below 50 years (564)	217(89·3)	261(81·3)	478(84·8)	6.84	0·009
Mean age (years ± SD)	37·8 ±10·1	40·0 ±10·9	39·0±10·6		
Ever married (534)	98(43·0)	190(62·1)	288(53·9)	19·2	<0·001
Unemployed (539)	101(43·5)	163(53·1)	264(49·0)	4·83	0·028
Has Dependents (514)	159(72·0)	187(63·8)	346(67·3)	3·78	0·052
Overall poor psychosocial assessment (536)	104(45·4)	152(49·5)	256(47·8)	0·88	0·348
Non-compliance (542)	33(14·2)	84(27·1)	117(21·6)	13·0	<0·001
Substance Abuse (524)	20(8·7)	69(22·2)	89(16·4)	17·7	<0·001
Paying patients (462)	197(96·6)	236(91·5)	433(93·7)	5·03	0·025
Worse 20% socioeconomic area in Cape Town (473)	143(68·8)	99(37·4)	242(51·2)	46·0	<0·001

*The χ^2^ and p-value are comparing race. All numbers are patient count unless indicated. SD Standard deviation

** Total number available data in the variable

^+^ Non-Blacks included Whites, Mixed ancestry and Asians

Patients assessed consisted of proportionally less blacks with non-compliance, substance abuse and ever married. This was statistically significant. There were also more blacks aged below 50 years of age (89vs 81%). Most blacks in the cohort resided from areas of low socioeconomic status.

### Socio-demographic and clinical characteristics

The socio-demographic, and clinical characteristics of patients are described in Tables 3 and 4.

**Table 3:** Socio-demographic characteristics of new patients presented to the renal assessment committee in Groote Schuur from 2008–2012.

Variable (N**)	Presentation periods			Χ^2^	p-value*
	2008–2009 N (%)	2010 N (%)	2011–2012 N (%)		
Male gender (564)	116(55·5)	64(62·1)	138(54·8)	1·72	0·42
Black race (564)	84(40·2)	44(42·7)	115(45·6)	1·38	0·50
Age below 50 years (564)	179(85·6)	88(85·4)	211(83·7)	0·37	0·83
Mean age (years ± SD)	38·4±10·5	39·8±10·1	39·3±10·8		
Ever married (534)	113(55·4)	54(55·1)	121(52·2)	0·52	0·77
Unemployed (539)	94(45·4)	51(51·0)	119(51·3)	1·72	0·42
Foreign nationality (564)	11(5·3)	3(2·9)	7(2·8)	2·2	0·33
Has Dependents (514)	128(65·6)	69(71·1)	149(67·1)	0·90	0·64
Overall poor psychosocial assessment (536)	90(45·0)	43(43·9)	123(51·7)	2·67	0·26
Non-compliance (542)	37(18·1)	20(20·0)	60(25·2)	3·43	0·18
Substance Abuse (524)	35(17·2)	19(19·0)	35(14·7)	1·08	0·58
Paying patients (462)	162(92·0)	80(93·0)	191(95·5)	1·99	0·37
Worse 20% socioeconomic area in Cape Town (473)	85(46·4)	48(54·5)	109(54)	2·7	0·27

*The χ^2^ and p-value are comparing the time periods. All numbers are patient count unless indicated.

** Total number available data in the variable

**Table 4:** Clinical characteristics of new patients presented for the renal replacement program in Groote Schuur hospital from 2008–2012.

Variable(N**)		Presentation periods			Χ^2^	p-value*
		2008–2009 N (%)	2010 N (%)	2011–2012 N(%)		
Major presentation (408)	Uremic	90(46·9)	31(43·7)	60(41·4)	8·5	0·198
	Fluid overload	73(38·0)	35(49·3)	71(49·0)		
Body mass index (526)	˃ 30kg/m^2^	36(18·2)	18(18·2)	198(13·5)	2·1	0·357
HIV status (564)	Positive	8(3·8)	11(10·7)	38(15·1)	16	<0·001
Hepatitis B surface antigen (564)	Positive	6(2·9)	7(6·8)	13(5·2)	2·7	0·255
Emergency dialysis required (563)	Yes	45(21·6)	20(19·4)	51(20·2)	0·2	0·885
Renal Disease (564)	Diabetes	30(14·4)	15(14·3)	36(14·3)	0·01	0·998
Number of co-morbid diseases (564)	None	132(63·2)	65(63·1)	154(61·1)	3·87	0·694
	1	65(31·1)	32(31·1)	75(29·8)		
	2	9(4·3)	4(3·9)	20(7·9)		
	3	3(1·4)	2(1·9)	3(1·2)		

HIV, Human immunodeficiency virus

*The χ^2^ and p-value are comparing the time periods. All numbers are patient count unless indicated

**The number cases available for the respective variable.

Blacks were slightly over represented compared to the recent Western Cape census (43·1% vs. 38·6%). There were 30% more males than females and the median age was < 40 years (range 13–60). Substance abuse and poor adherence were identified in 16·4% and 21·6% respectively.

Most patients were symptomatic with either uremia (N = 181, 44·4%) or fluid overload (N = 179, 43·9%) with a median serum creatinine concentration of 1005·5μmol/L. Despite being symptomatic, only 20% (N = 116) required emergency dialysis. The majority of patients had a BMI below 30kg/m^2^ (N = 441, 83·8%), and tested negative for Hepatitis B surface antigen (N = 538, 95·4%) and HIV (N = 214, 84·9%).

Except for HIV status (which increased over time) the three presentation periods were clinically comparable in terms of the presentation, BMI, hepatitis B status, emergency dialysis and presence of co-morbid diseases.

Hypertension (40·6%), diabetes (14·4%) or chronic glomerulonephritis (15·8%) were assessed as the underlying cause of ESKD. In a significant proportion (15·4%), no underlying etiology was found. HIVAN represented a small proportion (3·5%). Patients with diabetes were less likely to be accepted (9·2% vs 18·8%) than those with chronic glomerulonephritis (20% vs 12·2%, p = 0·008).

### Predicting acceptance into the renal replacement program

In multivariate analysis (Table 5), race, compliance to treatment, dependents, paying patients and co-morbid diseases were not predictors. Significant predictors of not being selected for RRT were age above 50 years, unemployment, poor psychosocial assessment, substance abuse and diabetes.

**Table 5:** Multivariate analysis of predictors of acceptance by the Renal Replacement committee among new patients presented in Groote Schuur Hospital from 2008–2012.

Predictor	B	Wald χ^2^	P	Odds Ratio	95% Confidence Interval	
Black Race	0·114	0·171	0·679	1·121	0·653	1·925
Age above 50 years	-1·367	10·398	0·001	0·255	0·111	0·585
Unemployment	-1·171	19·375	<0·001	0·310	0·184	0·522
Lack of Dependents	-0·161	0·334	0·563	0·851	0·494	1·469
Poor Psychosocial Assessment	-2·066	49·232	<0·001	0·127	0·071	0·226
Adherence History	0·625	2·911	0·088	1·868	0·911	3·827
Substance Abuse	-1·755	19·334	<0·001	0·173	0·079	0·378
Paying patient	0·155	0·069	0·793	1·167	0·368	3·705
Diabetes	-0·989	5·839	0·016	0·372	0·167	0·830
Co-morbid Disease/s	-0·359	1·585	0·208	0·699	0·400	1·221

### Effect of prioritization criteria

The introduction of prioritization criteria has not led to changes in outcomes of the assessments (Table 6). All category 1 patients received renal support as expected with the prioritization criteria. In contrast, 2 patients did not receive renal support prior to introduction of prioritization. Even if accepted, 27·4% (58/212) of category 2 patients did not get onto the program due to resource limitation. The prioritization criteria did not increase the proportion of category 2 patients who joined the program (p = 0·5). In totality, although the absolute number of patients accepted increased (Table 6), no change in the proportion of patients who were accepted was noted (p = 0·77). More HIV positive patients were accepted after introduction of the prioritization criteria (2% vs 13·9%, p = 0·006). This is because the new criteria defined the criteria for acceptance to the programme and an HIV positive transplant programme was developed. All other socio-demographic and clinical variables did not reveal any statistically significant differences between the assessment periods.

**Table 6:** Characteristics of Patients assessed by the renal replacement committee before and after the use of Prioritization Criteria (N = 564).

Variable	Presentation periods			Χ^2^	p-value**
	2008–2009 N (%)	2010 N (%)	2011–2012 N (%)		
Category 1	26(12·4)	15(14·6)	31(12·3)	0·45	0·98
Category 2	79(37·8)	39(37·9)	94(37·3)		
Category 3	104(49·8)	49(47·6)	127(50·4)		
Accepted patients	100(47·8)	45(43·7)	115(45·6)	0·52	0·77
Accepted HIV* Positive	8(3·8)	11(10·7)	38(15·1)	15·97	<0·001

*HIV Human immunodeficiency virus

**The χ^2^ and p-value evaluate the presentation periods.

## Discussion

This study describes the allocation outcome of patients assessed for the RRT program and explores the extent to which equity in selection is achieved. The average assessed candidate profiles a potential productive South African citizen i.e. 40 years male with dependents and classified as able to pay subsidized hospital bills (Table 3). Noting that over half (N = 304, 53·9%) were rejected by the committee, this represents a significant human resource loss to the South African society. These findings seem, at first instance, to negate the principle of utilitarianism. This refers to maximizing the benefit that the society will get from allocation of treatment. Utilitarianism is a criterion for selection of patients according to the current guideline.[9] However reference is made to transplantation rather than to dialysis treatment in these guidelines. The patients turned down had less favorable characteristics for kidney transplantation. This is unfortunately the cost of maintaining a RRT program in a resource limited setting.

The high cost of renal replacement therapy[16] combined with the rising burden of chronic kidney disease^8,15^has placed a strain on health systems globally. In Africa, the unmet need for managing ESKD is likely underestimated due to the obvious lack of data.[16] In countries with well-established government funded programs like South Africa, only about 12·5% of the dialysis need is met.[12] The success of the dialysis program in South Africa also stems from incorporating transplantation as a pre-requisite for treating patients.

The pressure for dialysis slots is likely to intensify giving the growing population, and the lack of provision for new facilities. The population in South Africa has increased by 10 million in 18 years yet there was an addition of only 2 dialysis units in the public sector.[4] In this audit the number of new patients presented to the Committee between 2008 and 2012 increased. This, coupled by a decrease in the number of transplants will continue to place strain on the available dialysis slots. During this period only 46% (N = 260) of 564 patients could be accepted onto the program. This number excludes patients that were never presented to the program e.g. diabetic patients over 50 years of age, patients older than 60 years and patients living with HIV with uncontrolled viral load. There is thus a major underestimation of patients requiring dialysis. A similar proportion of patients (47%) were accepted in an earlier series at Tygerberg Hospital, another Western Cape hospital.[12]

Even though in both series age and employment status were both prominent decisive factors, Moosa et.al., had more whites being selected than blacks.[12] This is in contrast to our results which document more blacks being accepted than non-blacks in univariate analysis. However in multivariate analysis, race was not a predictor for acceptance onto treatment. One may hypothesize that the disparities in access to health have improved over the years in alignment with the government’s policy to provide health care for all. The South African renal registry data support this hypothesis as the proportion of black South Africans on dialysis has increased from 31·2% in 1994 to 51·2% in 2012.[4] However the growth of patients recorded in the registry is largely driven by the insured population leaving many unemployed Black South Africans lacking access to dialysis especially in centres outside the major urban areas. Despite this, our results show an encouraging trend 20 years after demise of the apartheid system. Our results may reflect the similarities in socio-economical characteristics among the uninsured South Africans attending a state sponsored hospital. Disparities in access to health, particularly in those uninsured is not a local phenomenon but rather a global concern.[8,16]

Disparity in selecting patients based on employment status has been previously reported in South Africa.[12] Unemployment was an important predictor of being rejected from the RRT program at Groote Schuur. This is no longer a discriminatory factor in the current Western Cape guidelines. Despite this, the guidelines may still negatively affect unemployed candidates. Acceptance onto the program requires evidence of financial means to regularly arrange for transport to the renal unit, which is part of the criteria. About two thirds (68% of 257 accepted patients) were employed in our cohort. In contrast to our findings only 11·4% of 290,252 patients being prepared for ESKD care in the United States were employed.[17]

Other socio-demographic factors such as gender, marital status, nationality and area of residence were not predictors of acceptance among presented patients. This adds strengths to the use of current guidelines which do not discriminate against these groups.

Inadequate access to health care probably explains another finding in our audit. The majority of patients presented to our RRT program were in advanced disease stages, forty four percent (N = 181) had uremic symptoms and more than half had a serum creatinine >1000μmol/L. Patients who are socioeconomically disadvantaged tend to present late to a nephrologist[18,19] and have less than optimal outcome on treatment.[20] This is consistent with the advanced presentation of patients in our series, as over half of the presented patients 51% (N = 242) came from the lowest socioeconomic areas of Cape Town. This work indirectly reveals the challenges in the current health system of the Western Cape.

The difficulty in early diagnosis was also seen in HIV positive patients. In our cohort, HIV positive patients were under-represented. HIV positive patients accounted for only 10% of all new patients presented to the selection committee despite a high prevalence of HIVAN. For example a study at Groote Schuur showed that 44% of all patients with nephrotic syndrome had HIVAN with severely impaired renal function.[21] Under representation of HIV positive patients arises from the fact that most had outright exclusion criteria because of uncontrolled HIV disease. Early diagnosis and management of HIV would allow more HIV positive candidates to be considered for dialysis and transplantation, and, notably, HIV status was not a predictor of acceptance onto the program among new patients presented.

### Study limitations

The retrospective study design depended on available reports which had some missing data. These results may not be generalized to the whole South African population because there is a significant dialysis population in the private sector.

## Conclusion

Twenty years after the end of apartheid, South Africa has made improvements in disparities to access of dialysis despite resource challenges. In the setting of resource limitation, rationing of dialysis becomes unavoidable in running a sustainable program. Efforts to allocate more resources should continue in view of the loss of young and potentially productive life. Advanced presentation of patients with ESKD represents challenges in early diagnosis and referral in the current system.
